# Correction: Electrochemical detection of chlorogenic acid in green coffee beans by a carbon paste electrode modified with MWCNTs and Cr-MOF

**DOI:** 10.1039/d5ra90142a

**Published:** 2025-12-15

**Authors:** Maryam Khayatkashani, Safaa Abdulkadhim Almansarawi, Ebrahim Honarmand, Fateme Sadat Sadeghi, Ali Ehsani, Layth S. Jasim, Masoud Salavati-Niasari

**Affiliations:** a Advanced Medical Pharma (AMP-Biotec), Biopharmaceutical Innovation Centre Via Cortenocera San Salvatore Telesino BN Italy; b Department of Chemistry, University of Sumer, College of Education Thi-Qar Iraq; c Department of Chemistry, Faculty of Science, University of Qom Qom Iran e.honarmand@qom.ac.ir; d Department of Chemistry, College of Education, University of Al-Qadisiyah Diwaniyah Iraq; e Institute of Nano Science and Nano Technology, University of Kashan P.O. Box 87317-51167 Kashan Iran salavati@kashanu.ac.ir +98 31 55913201 +98 31 55912383

## Abstract

Correction for ‘Electrochemical detection of chlorogenic acid in green coffee beans by a carbon paste electrode modified with MWCNTs and Cr-MOF’ by Maryam Khayatkashani *et al.*, *RSC Adv.*, 2025, **15**, 41314–41329, https://doi.org/10.1039/D5RA05186J.

The authors regret that both Fateme Sadat Sadeghi and Ali Ehsani were incorrectly associated with affiliation a in the original article. The correct affiliation information is as shown here, whereby Fateme Sadat Sadeghi and Ali Ehsani are associated with affiliation c (Department of Chemistry, Faculty of Science, University of Qom, Qom, Iran).

The Royal Society of Chemistry apologises for these errors and any consequent inconvenience to authors and readers.

